# Atrial Fibrillation Catheter Ablation among Cancer Patients: Utilization Trends and In-Hospital Outcomes

**DOI:** 10.3390/jcm13051318

**Published:** 2024-02-26

**Authors:** Gilad Margolis, Ofir Goldhaber, Mark Kazatsker, Ofer Kobo, Ariel Roguin, Eran Leshem

**Affiliations:** 1 Division of Cardiovascular Medicine, Hillel Yaffe Medical Center, The Ruth and Bruce Rappaport Faculty of Medicine, Technion, Haifa 38100, Israel; o.goldhaber@gmail.com (O.G.); mark.kazatsker@gmail.com (M.K.); ofermkobo@gmail.com (O.K.); aroguin@technion.ac.il (A.R.); dr.eranleshem@gmail.com (E.L.); 2 Cardiac Electrophysiology Unit, Hillel Yaffe Medical Center, Hadera 38100, Israel

**Keywords:** atrial fibrillation, catheter ablation outcomes, cancer

## Abstract

**Background:** Atrial fibrillation (AF) catheter ablation in cancer patients has been evaluated in very few studies. We aimed to investigate utilization trends and in-hospital outcomes of AF catheter ablation among cancer patients in a large US inpatient registry. **Methods:** Utilizing the National Inpatient Sample (NIS) database, patients who underwent AF catheter ablation between 2012 and 2019 were identified. Sociodemographic, clinical data, in-hospital procedures and outcomes were collected. Baseline characteristics and in-hospital outcomes were compared between patients with and without cancer. **Results:** An estimated total of 67,915 patients underwent AF catheter ablation between 2012 and 2019 in the US. Of them, 950 (1.4%) had a cancer diagnosis. Patients with a cancer diagnosis were older and had higher Charlson Comorbidity Index, CHA2DS2-VASc and ATRIA bleeding indices scores. A higher rate of total complications was observed in cancer patients (10.5% vs. 7.9, *p* < 0.001), driven mainly by more bleeding and infectious complications. However, no significant differences in cardiac or neurological complications as well as in-hospital mortality rates were observed and were relatively low in both groups. **Conclusions:** AF catheter ablation in cancer patients is associated with higher bleeding and infectious complication rates, but not with increased cardiac complications or in-hospital mortality in a US nationwide, all-comer registry.

## 1. Introduction

Atrial fibrillation (AF) is the most common sustained arrhythmia affecting 2–4% of the general population [1]. Cancer patients have an increased risk for developing AF, varying according to cancer type and stage [2,3]. Conversely, elevated cancer risk was observed among patients presenting with new-onset AF [4]. The exact pathophysiological mechanisms leading to AF in cancer patients are not yet fully understood. Suggested underlying mechanisms include a similar risk factor profile for both AF and cancer development, inflammatory and paraneoplastic processes, autonomic nervous system imbalance or, less commonly, direct metastatic invasion of the tumor to the heart and surrounding tissues [5]. In addition, both medical and surgical cancer treatments were implicated as predisposing factors for AF development [6,7]. A recent analysis of a very large pharmacovigilance database identified 19 anticancer drugs, ¾ of which are used in hematologic malignancies, significantly associated with AF development [8]. The mechanisms by which anticancer drugs induce AF are not established. Proposed mechanisms include direct or indirect effects on cardiac ion channels, affecting action potential duration; oxidative stress and activation of inflammation pathways, which can cause myocardial fibrosis and atrial remodeling, promoting AF [9]. Surgical therapy for cancer was also implicated with AF development. The incidence of perioperative AF in cancer surgery ranges from 4 to 5% for cholecystectomy, 9 to 11% for esophagectomy, and 6 to 28% for lung resection [6]. However, the AF incidence in the general population undergoing noncardiac surgery ranges between 10 and 20%, attributed to transient perioperative autonomic imbalance and inflammation [10]. The direct effect of cancer on perioperative AF risk is yet to be determined.

Coexisting AF in cancer patients has been associated with increased risk for both bleeding and thromboembolism [6]. Current guidelines recommend that before deciding on anticoagulation for individuals with cancer, available risk stratification tools such as the CHA_2_DS_2_-VASc score (congestive heart failure, hypertension, age ≥ 75 (2 points); diabetes mellitus, stroke (2 points); vascular disease, age 65–74 years, sex category (female)) and bleeding risk scores should be taken into account along with cancer stage, drug–drug interactions and patient preferences among other considerations [5].

Rhythm control for symptomatic AF in cancer patients portends a therapeutic challenge. Significant drug–drug interactions with concomitant anticancer therapy may preclude the use of antiarrhythmic drugs (AADs) [11]. Catheter ablation for AF is an effective and safe treatment strategy for symptomatic patients who do not benefit from AADs [1]. However, to date, very few studies have evaluated AF catheter ablation among cancer patients [12,13,14,15,16,17]. Current guidelines and expert opinion positions papers do not provide clear recommendations for AF rhythm control in cancer patients, and specific recommendations for catheter ablation are lacking [1,5,18,19].

In this study, we aimed to analyze trends in utilization and in-hospital outcomes of AF catheter ablation procedures among cancer patients using the US National In-Patient Sample (NIS) database.

## 2. Materials and Methods

### 2.1. Data Source

The data were drawn from the NIS, the Healthcare Cost and Utilization Project, and the Agency for Healthcare Research and Quality. The NIS database includes only non-identified data. Therefore, this study was deemed exempt from institutional review by the local Human Research Committee. The NIS is the largest collection of all-payer data on inpatient hospitalizations in the United States. The data set represents an ≈20% stratified sample of all inpatient discharges from US hospitals [20]. This information includes patient-and hospital-level factors such as patient demographic characteristics, primary and secondary diagnoses and procedures, comorbidities, length of stay (LOS), hospital region, hospital teaching status, hospital bed size, and cost of hospitalization. National estimates can be calculated using the patient and hospital level sampling weights that are provided by the Healthcare Cost and Utilization Project.

For the purpose of this study, we obtained data for the years 2012 to 2019. The International Classification of Diseases, Tenth Revision, Clinical Modification/Procedure Coding System (ICD-10-CM/PCS) was fully implemented from the last quarter of 2015 and thereafter for reporting diagnoses and procedures in the NIS database during the study period.

From 2012 to 2015 (3rd quarter), hospitalizations were analyzed using the ICD-9-CM/PCS coding system and, subsequently, with the ICD-10-CM/PCS coding system. For each index hospitalization, the database provides a principal discharge diagnosis and a maximum of 39 additional diagnoses, in addition to a maximum of 25 procedures.

### 2.2. Study Population and Variables

We identified patients aged ≥18 years who had a primary diagnosis of atrial fibrillation using ICD-10-CM codes: I480, I481, I482, I4891 or ICD-9-CM code: 427.31 and underwent ablation using ICD-10-PCS codes: 02583ZZ, 02563ZZ, 02573ZZ, 025T3ZZ, 025S3ZZ or ICD-9-CM code: 37.34. Using ICD-10-CM and ICD-9-CM codes (provided in detail in Table S1) we identified and excluded patients who had any of the following diagnoses: supraventricular tachycardia, atrioventricular nodal tachycardia, ventricular tachycardia, ventricular fibrillation, atrial flutter, ventricular or atrial premature beats, Wolff–Parkinson–White syndrome. To avoid inclusion of patients undergoing atrioventricular junction ablation only (as a “pace and ablate” strategy), we excluded patients with a cardiac implantable electronic device (CIED) in situ. We also excluded patients who underwent CIED implantation during their hospitalization to avoid attributing their complications to the ablation procedure. A similar data extraction methodology was previously utilized to identify patients undergoing AF ablation in the NIS registry [21,22].

The following patient demographics were collected from the database: age, sex, and ethnicity. ICD-10-CM codes and ICD-9-CM codes (Table S1) were used to identify different comorbidities, including diabetes, hypertension, chronic heart failure, chronic kidney disease, obstructive sleep apnea, obesity, chronic pulmonary disease, anemia, hypertrophic cardiomyopathy, and valvular heart disease.

For the purposes of calculating the Charlson–Deyo comorbidity index, additional comorbidities were identified from the database using ICD-10-CM codes and ICD-9-CM codes. The Charlson–Deyo comorbidity index is a modification of the Charlson comorbidity index, containing 19 comorbidity conditions with differential weights, with a total score ranging from 0 to 38 [23,24,25]. Detailed information on the Charlson–Deyo comorbidity index is provided in Table S2. Higher Charlson–Deyo comorbidity index scores indicate a greater burden of comorbid diseases and are associated with increased risk of death within 1 year after admission. The index has been used extensively in studies from administrative databases, with proven validity in predicting short-and long-term outcomes [26,27,28]. In addition, the CHA_2_DS_2_-VASc score was calculated for each patient. We calculated ATRIA bleeding risk scores (anemia, severe kidney disease, age ≥ 75, history of hemorrhage and hypertension) for each patient based on information available in the NIS dataset. We were not able to calculate the more commonly used HAS-BLED score, as the NIS does not provide information regarding patients’ blood tests and medications. The ATRIA and HAS-BLED bleeding risk scores were recently evaluated among cancer patients, with comparable performance in predicting any and major bleeding events [29].

We obtained information on the presence of a known malignancy for each patient, based on ICD-10-CM codes and ICD-9-CM codes outlined in Table S3. Malignancies were categorized into two groups: hematologic malignancies and solid malignancies.

The primary outcome in this study was in-hospital complications including death. Secondary outcomes included sub-groups of complications, as well as LOS and total charges. Using ICD-10-CM/PCS codes and ICD-9-CM/PCS codes (Table S1), the following in-hospital complications were identified: hemopericardium, tamponade, acute heart failure, cardiogenic shock, cardiac arrest, periprocedural hemorrhage/hematoma requiring transfusion, vascular injury (e.g., arteriovenous fistula, aneurysm or pseudoaneurysm), post-procedure respiratory failure, periprocedural diaphragmatic disorder, periprocedural stroke and sepsis.

### 2.3. Statistical Analysis

Frequencies and proportions of the different demographic, clinical, and hospital-related variables were calculated and weighted to reflect national estimates using discharge sample weights provided by the NIS [27]. These estimates were compared according to cancer/non-cancer grouping using the Pearson’s chi-square test and independent-samples *t*-test for categorical variables and continuous variables, respectively. Generalized linear models were used to analyze annual trends. For all statistical analyses, we utilized SPSS^®^ software version 23 (IBM Corp., Armonk, NY, USA). A *p*-value < 0.05 was considered statistically significant.

## 3. Results

### 3.1. Baseline Characteristics of the Study Population

A total of 13,583 hospitalizations for AF ablation across the United States during the study period were included in the analysis. After implementation of the weighting method, these represented an estimated total of 67,915 hospitalizations for AF ablation. Most patients (61%) were men, and the mean age of the cohort was 64.4 + 10.9 years. Of the total estimated cohort, 950 patients (1.4%) had a diagnosis of cancer.

Baseline characteristics of the study population according to cancer/non-cancer grouping are presented in detail in Table 1. Older age, male gender, heart failure, chronic kidney disease and anemia were more prevalent in the cancer group. In addition, cancer patients had both higher CHA2DS2-VASc (2.72 ± 1.36 vs. 2.32 ± 1.45, *p* < 0.001) and ATRIA bleeding scores (1.91 ± 1.86 vs. 1.21 ± 1.37, *p* < 0.001; Table 1).

**Figure 1:**
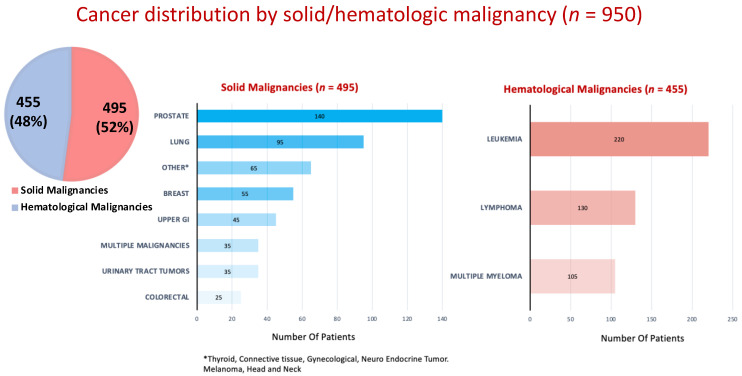
Cancer distribution by solid/hematologic malignancy.

Among cancer patients who underwent AF ablation during the study period, 495 (52%) had a solid malignancy, while 455 (48%) had a hematologic malignancy (Figure 1). Among solid malignancies, prostate cancer and lung cancer were the most frequent, and leukemia was the most frequent of the hematologic malignancies (Figure 1).

**Figure 2:**
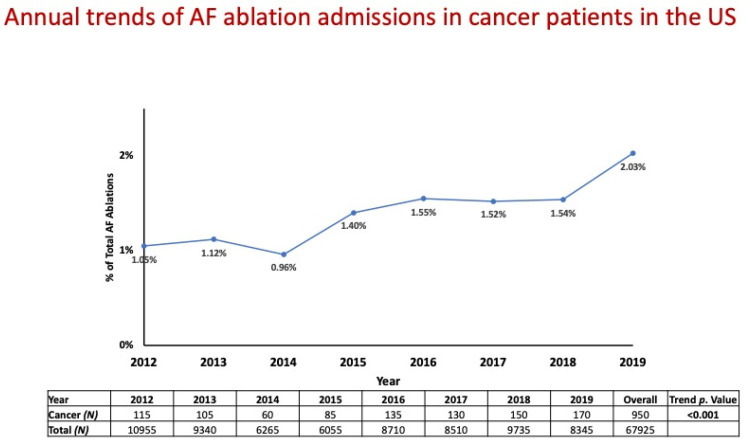
Annual trend in AF catheter ablations for cancer patients between 2012 and 2019.

The annual trend in AF catheter ablations for cancer patients gradually increased from 1.05% (*n* = 115) in 2012 to 2.03% (*n* = 170) in 2019 (*p* < 0.001; Figure 2).

### 3.2. In-Hospital Course and Outcomes by Cancer/Non-Cancer Groups

In-hospital complications occurred in 5400 patients (8%) admitted for AF ablation. Compared with those without cancer, patients who had a cancer diagnosis had a higher unadjusted rate of total in-hospital complications (10.5% vs. 7.9%, *p* < 0.001). The higher complication rate in the cancer group was driven by an increased rate of bleeding (3.2% vs. 1.8%. *p* = 0.002) and infectious complications (2.1% vs. 0.8%, *p* < 0.001; Table 2). No significant differences were observed in cardiac complications, periprocedural stroke and in-hospital mortality rates, which were low in both groups (Table 2). The average LOS in the hospital was longer in cancer patients compared with non-cancer patients (4.91 + 5.8 vs. 2.75 + 3.05, Table 2).

**Figure 3:**
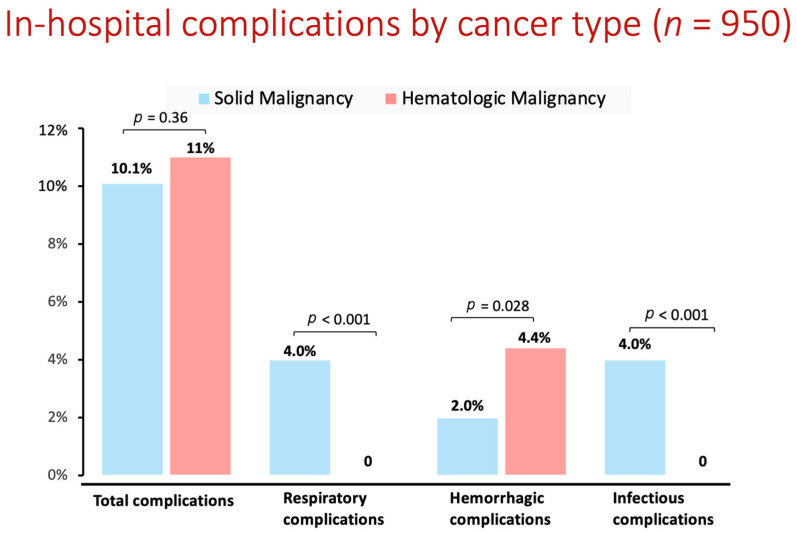
In-hospital complications by cancer type.

Analysis of in-hospital complications by cancer type showed no significant difference in the total complication rate between patients with solid or hematologic malignancies (10.1% vs. 11%, *p* = 0.36). However, patients with hematologic cancer had a significantly higher rate of bleeding complications, while those with solid cancer had higher rates of post-procedural respiratory failure and infectious complications (Figure 3).

## 4. Discussion

Using data from the NIS, the largest all-payer inpatient database in the United States, we analyzed a weighted total of 67,915 catheter ablation procedures for AF, between January 2012 and December 2019. Real-world nationwide data showed that 950 patients (1.4%) undergoing AF ablation had a cancer diagnosis. Annual trend analysis showed a gradual and significant increase in AF catheter ablations for cancer patients between 2012 and 2019. Cancer patients undergoing AF catheter ablation were older, had more comorbidities and elevated thrombotic as well as bleeding risks, reflected by higher CHA2DS2-VASc and ATRIA bleeding indices, compared to their non-cancer counterparts. However, despite a higher rate of total complications among cancer patients, driven by infectious and bleeding complications, no significant differences were observed in cardiac complications, periprocedural stroke or in-hospital mortality rates.

Current guidelines recommend pursuing rhythm control in symptomatic AF patients [1]. However, this may be challenging in cancer patients who also have AF. Maintaining sinus rhythm with anti-arrhythmic medications may involve significant drug–drug interactions with anti-cancer agents [11]. There is scarce data on the efficacy of catheter ablation in this population. Nevertheless, observational data suggests that catheter ablation is equally effective in patients with or without a cancer history [15,16].

Very few studies assessed the safety of AF catheter ablation in cancer patients with discrepant results. Giustozzi et al. showed higher risk for clinically relevant periprocedural bleeding compared to non-cancer patients in 21 cancer patients who underwent AF catheter ablation [14]. In two other studies, the frequency of periprocedural bleeding was similar between cancer and non-cancer patients [15,16]. Of note, different periprocedural anticoagulation protocols were utilized in these studies, as no such protocol was ever verified in cancer patients. In our analysis, the most common periprocedural complications were vascular/bleeding complications in both cancer and non-cancer patients. Previous studies also showed that the most prevalent complications of atrial fibrillation ablations are vascular injuries, which could be potentially mitigated by ultrasound guided vascular access [30,31]. This could be particularly pertinent for cancer patients, which, in our study, had a higher rate of bleeding complications compared to non-cancer patients. We do not have data on the anticoagulation regimen given during and after the ablation in our cohort. Notably, the excess bleeding we observed was in patients with hematologic malignancies, which constituted almost half of patients, a higher proportion than in previous studies [12,13,14,15,16]. In a retrospective analysis of patients undergoing percutaneous coronary interventions, leukemia diagnosis was associated with higher periprocedural bleeding complications [32]. Whether hematologic cancer patients are at higher risk for bleeding complications in AF ablations needs to be evaluated in future studies.

Infectious outcomes were not reported in prior studies of AF ablation in cancer patients, who are potentially immunocompromised, due to both disease and treatment [5]. In our study, we report, for the first time, a non-negligible rate of periprocedural infectious complications in cancer patients (2.1%), significantly higher than in non-cancer patients (0.5%). To date, there is no recommendation regarding periprocedural antibiotic prophylaxis for patients undergoing AF ablation [33]. Guidelines for vascular and interventional radiology procedures recommend against antibiotic prophylaxis in patients undergoing cardiac procedures (e.g., coronary angioplasty), but give a IIb indication for antibiotic prophylaxis in patients undergoing solid tumor radiofrequency ablation, with the rationale being that thermal injury during this procedure may create a hospitable environment for bacteria [34]. Whether cancer patients undergoing AF ablation need periprocedural antibiotic prophylaxis should be evaluated in future studies.

Recently, Thotamgari et al. [17] evaluated AF ablation procedure outcomes in 750 cancer patients identified in the NIS database between the years 2016 and 2019. Utilizing propensity score matching technique, they reported a higher in-hospital mortality rate in cancer patients compared with non-cancer patients (2% vs. 0.7%). Importantly, patients with in situ CIEDs were not excluded from their analysis. There are no specific ICD 9/10 codes for AV node ablation, so it is possible that such procedures were included in their analysis. As AV node ablation is usually reserved for the older and sicker patients [1,19], the possibility of selection bias resulting in an unsound signal of excess mortality is conceivable. This could potentially explain their discrepant results with ours as well as with previous studies [12,13,14,15,16].

## 5. Limitations

Several limitations should be acknowledged. The NIS database is a retrospective administrative database that contains discharge-level records and, as such, is susceptible to coding errors. A lack of information about patients’ cancer status in the NIS prevents us from distinguishing between patients with active cancer and patients with inactive disease, as well as concurrent anti-neoplastic treatment.

Additionally, the NIS database lacks granularity regarding the type of ablation performed (e.g., pulmonary vein isolation only or with additional substrate modification) or the type of energy used during ablation, such as thermal energy (e.g., cryoablation, radiofrequency) or nonthermal energy (e.g., pulse field ablation). However, to date, no major safety differences were observed between different ablation approaches or energy delivery methods [35,36,37]. Additionally, no data were available regarding the number of pulmonary veins isolated during each procedure.

In addition, our study included a relatively small sample of cancer patients who underwent AF ablation procedures. Nonetheless, with 950 cancer patients, this is the largest cohort reported to date in this population [12,13,14,15,16,17].

We could only capture events that occurred in the same index hospitalization. The NIS does not include any follow-up data, nor does it include the exact timing of in-hospital outcomes during hospitalization (e.g., periprocedural stroke timing). All data are intentionally non-identified. The NIS database also does not include detailed information about patients’ clinical characteristics, medication, blood tests, and so on. We had no data about anticoagulation regimens during the peri-procedural period. These limitations are counterbalanced by the real-world, nationwide nature of the data, lack of selection bias, and absence of reporting bias introduced by selective publication of results from specialized centers. These results should not infer causation of periprocedural risk by malignancy, but merely present a real-world association that requires randomized trials for validation.

## 6. Conclusions

The number of cancer patients undergoing AF catheter ablation procedures in the US increased steadily during the study period of 2012–2019. AF catheter ablation for cancer patients was associated with higher bleeding and infectious complication rates, but not with increased cardiac complications or in-hospital mortality in a nationwide, all-comer registry. These findings suggest that catheter ablation is a safe treatment modality for cancer patients who also experience AF.

## Figures and Tables

**Table 1:** Baseline characteristics of patients undergoing AF catheter ablation.

Variable	Non-Cancer Patients	Cancer Patients	*p* Value
	(*n* = 66,965)	(*n* = 950)	
Age (years)	64 ± 11	69 ± 9	<0.001
Female	26,140 (39%)	310 (33%)	<0.001
White ethnicity	57,250 (86%)	840 (88%)	0.006
Diabetes mellitus	13,945 (21%)	225 (24%)	0.017
Hypertension	38,265 (57%)	500 (53%)	0.003
Heart failure	16,585 (25%)	370 (39%)	<0.001
Valvular heart disease	8385 (13%)	140 (15%)	0.023
Chronic kidney disease	6655 (10%)	170 (18%)	<0.001
Chronic Pulmonary Disease	9875 (15%)	195 (21%)	<0.001
Obesity (BMI > 30)	15,430 (23%)	215 (23%)	0.398
Obstructive sleep apnea	14,985 (22%)	190 (20%)	0.044
Prior stroke	1155 (1.7%)	15 (1.6%)	0.414
Prior myocardial infarction	3760 (6%)	60 (6%)	0.003
Hypertrophic cardiomyopathy	610 (0.9%)	None	NA
Peripheral artery disease	2670 (4%)	55 (6%)	0.003
Anemia	4000 (6%)	195 (21%)	<0.001
Charlson comorbidity index ≥ 2	15,700 (24%)	900 (95%)	<0.001
CHA_2_DS_2_-VASc score	2.32 ± 1.45	2.72 ± 1.36	<0.001
0–1	21,455 (32%)	185 (19%)	<0.001
≥2	45,510 (68%)	765 (81%)	
ATRIA bleeding score	1.21 ± 1.37	1.91 ± 1.86	<0.001
0–1	51,130 (76%)	520 (55%)	<0.001
≥2	15,835 (24%)	430 (45%)	
Income percentile			
0–25	13,700 (21%)	150 (16%)	0.001
26–50	15,540 (23%)	255 (27%)	
51–75	17,610 (26%)	240 (25%)	
76–100	20,115 (30%)	305 (32%)	
Teaching hospital	55,000 (82%)	770 (81%)	0.206
Hospital region			
South/west	37,900 (57%)	515 (54%)	0.075
Midwest/northeast	29,065 (43%)	435 (46%)	

**Table 2:** In-hospital diagnoses and procedures of patients undergoing AF catheter ablation with/without cancer.

Variable	Non-Cancer Patients	Cancer Patients	*p* Value
	(*n* = 66,965)	(*n* = 950)	
Total complications	5300 (7.9%)	100 (10.5%)	0.002
Cardiac complications			
Cardiogenic shock	80 (0.1%)	None	NA
Cardiac arrest	100 (0.1%)	None	NA
Acute heart failure	445 (0.7%)	≤10	NA
Hemopericardium/cardiac tamponade	1085 (1.6%)	≤10	NA
Vascular complications			
Hemorrhage/hematoma/blood transfusion	1230 (1.8%)	30 (3.2%)	0.002
Vascular injury	1065 (1.6%)	15 (1.6%)	0.557
Respiratory complications			
Post-procedural respiratory failure/invasive ventilation > 24 h	1125 (1.6%)	20 (2.1%)	0.188
Diaphragmatic disorders	190 (0.2%)	None	NA
Neurologic complications (stroke)	95 (0.1%)	≤10	NA
Infectious complications (bacteremia, sepsis)	530 (0.8%)	20 (2.1%)	<0.001
Length of stay	2.75 ± 3.05	4.91 ± 5.8	<0.001
In-hospital mortality	165 (0.2%)	≤10	NA

NA = not applicable.

## Data Availability

The national database data used for this study, analytic methods, and study materials will not be made available to other researchers for purposes of reproducing the results or replicating the procedure because of restrictions on the sharing of data in the Healthcare Cost and Utilization Project Data Use Agreement. The NIS database is publicly available for purchase, and the transparent and detailed methods that we have described make it possible for anyone who wishes to do so to replicate this study and reproduce our results.

## Supplementary Materials

The following supporting information can be downloaded at: https://www.mdpi.com/article/10.3390/jcm13051318/s1, Table S1: ICD-10 and ICD-9 CM/PCS codes used in data extraction; Table S2: ICD-10 CM and ICD-9 CM codes for conditions incorporated in Deyo-CCI, and scoring system used to compute CCI scores; Table S3: ICD-10 CM and ICD-9 CM codes for primary cancer site diagnosis.
