# Supplementary material for: Atrial Fibrillation Catheter Ablation among Cancer Patients: Utilization Trends and In-Hospital Outcomes
Source: J Clin Med. 2024 Feb 26;13(5):1318. doi: 10.3390/jcm13051318 (PMC10932365; doi:10.3390/jcm13051318)
Supplement: Supplementary file 1 [file jcm-13-01318-s001.zip › jcm-2855571-supplementary.pdf]

Table S1: ICD-10 and ICD- 9 CM/PCS codes used in data extraction

ICD-10 CM/PCS codes	ICD-9 CM/PCS codes	Condition/Procedure
**Codes for Inclusion Criteria.**		
I480, I481, I482, I4891	427.31	Atrial Fibrillation Primary Diagnosis
02583ZZ, 02563ZZ, 02573ZZ, 025T3ZZ, 025S3ZZ	37.34	Catheter Ablation Primary Procedure
**Codes for Exclusion Criteria.**		
I47.0, I47.1, I47.9	427.0,427.89,426.7,426.89	Supraventricular tachycardia
I47.2	427.1	Ventricular tachycardia
I48.3, I48.4, I48.9	427.32	Atrial flutter
I456	426.7	Preexcitation
02HK3JZ, 02HK4JZ, 0JH605Z, 0JH635Z, 0JH606Z, 0JH636Z, 02H64JZ, 02H63JZ, 0JH604Z, 0JH634Z, 0JH804Z, 0JH834Z, 0JH805Z, 0JH835Z, 0JH806Z, 0JH836Z	00.50, 00.52, 00.53, 37.71, 37.72, 37.73, 37.74, 37.75, 37.76, 37.77, 37.78, 37.79, 37.80, 37.81, 37.82, 37.83, 37.85, 37.86, 37.87, 37.89.	Pacemaker implantation
02HK3KZ, 02HK4KZ, 0JH608Z, 0JH638Z, 0JH808Z, 0JH838Z	37.94, 37.95, 37.96, 37.97, 37.98	ICD implantation
0JH607Z, 0JH637Z, 0JH609Z, 0JH639Z, 02H43JZ, 02H44JZ, 02H40JZ, 02H40NZ, 02H43NZ, 02H44NZ, 0JH807Z,	00.51, 00.54.	Cardiac resynchronization therapy implantation

0JH837Z, 0JH809Z, 0JH839Z		
Z95.0, Z95.810	V45.01, V45.02	History of Pacemaker/ICD
02580ZZ	37.33	Open surgical ablation
**Codes for baseline characteristics and comorbidities not included in Deyo-CCI**		
I10	401.0, 401.1, 401.9	Hypertension
G4730, G4733	327.23	Obstructive sleep apnea
E66.01, E66.2, E66.09, E66.1, E66.8, E66.9, Z68.4, Z68.3	278.0x	Obesity
D50.x, D51.x, D52.x, D53.x, D64.x	280.x, 281.x, 285.0, 285.8, 285.9	Anemia
I42.1, I42.2	425.1x	Hypertrophic Cardiomyopathy
I351, I350, I352, I358, I359, I340, I341, I342, I348, I349, I360, I361, I362, I368, I369, I050, I051, I052, I058, I059, I060, I061, I062, I068, I069, I070, I071, I072, I078, I079, I080, I081, I082, I083, I088, I089	424.0, 424.1, 424.2, 424.3, 394.x, 395.x, 396.x	Valvular heart disease
I60.x, I61-2.x, H356, H313, H431, H0523, I8501, I8511, K2901, K2921, K2931, K2941, K2951, K2961, K2971, K2981, K2991, K920, K921, K922, K5701, K5721, K5731, K5733,	430.x, 362.81, 376.32, 379.23, 578.x, 537.84, 719.1x, 729.92	Any Prior Hemorrhage Diagnosis

K5741, K5781, K625, M250, M7981, K661		
**Procedural complications related ICD codes - Vascular Complications.**		
K916, K9184, I974, G9752, I97618, I97618, N99821 30233H, 30233N	285.1, 998.11, 998.12, 990.x	Hemorrhage/hematoma/blood transfusion
T81719A. T81718A, T81711A, T81710A, I975, I976, S750, S751, S651, I770, I724.	997.2x, 868.40, 997.02, 998.2x, 900.x, 901.x, 902.x, 903.x, 904.x, 447.x, 393.1x, 394.1x, 394.9x, 395.2x, 395.3x, 395.6x, 395.7x, 395.8x, 395.9x, 397.9x, 447.0, 442.3	Vascular injury
**Procedural complications related ICD codes - Cardiac Complications.**		
T8111XA, T8110XA	998.01	Shock
I9712x, I977x	427.5	Cardiac Arrest
I9711x, I9713x, I9719x	428.21, 428.31, 428.41	Acute Heart Failure
I312	423.0x	Hemopericardium
I314	423.3x	Cardiac temponade
**Procedural complications related ICD codes - Respiratory Complications.**		
J9582, J9588, J9589 5A1935Z, 5A1945Z, 5A1955Z, 0B110F4, 0B113F4	518.51, 518.53, 967.x	Post procedure respiratory failure or Intubation >24 hrs
J986	519.4x	Diaphragmatic disorder

**Procedural complications related ICD codes -<br>Neurologic Complications.**		
I9781x, I9782x	997.00, 997.01, 997.02, 435.9x	Stroke
**Procedural complications related ICD codes –<br>Infectious Complications.**		
T80211A, T8149XA, T8144XA, T8112XA, A40x, A41x, R6520, R6521	780.60, 780.62, 995.91, 995.92, 998.02, 790.7x, 997.32, 038.x	Sepsis

Table S2: ICD-10 CM and ICD-9 CM Codes for conditions incorporated in Deyo-CCI, and scoring system used to compute CCI scores

ICD-10 CM codes	ICD-9 CM codes	Condition	Score
I21.x, I22.x, I25.2	410.x, 412.x	Myocardial infarction	1
I11.0, I13.0, I13.2, I25.5, I42.0, I42.5-I42.9, I43.x, I50.x, P29.0	428.x	Congestive heart failure	1
I70.x, I71.x, I73.1, I73.8, I73.9, I77.1, I79.0, I79.1, I79.8, K55.1, K55.8, K55.9, Z95.8, Z95.9	443.9, 758.4, 444.x, 441.x, V43.4x	Peripheral vascular disease	1
G45.x, G46.x, H34.0x, H34.1x, H34.2x, I60.x-I68.x	430.x, 431.x, 432.x, 433.x, 434.x, 435.x, 436.x. 437.x, 438.x	Cerebrovascular disease	1
F01.x-F03.x, F04, F05, F06.1, F06.8, G13.2, G13.8, G30.x, G31.0x, G31.1, G31.2, G91.4, G94, R41.81, R54	290.x	Dementia	1
J40.x-J47.x, J60.x-J67.x, J68.4, J70.1, J70.3	490.x, 491.x, 492.x, 493.x, 494.x, 495.x, 496.x, 500.x, 501.x, 502.x, 503.x, 504.x, 505.x	Chronic pulmonary disease	1
M05.x, M06.x, M31.5, M32.x-M34.x, M35.1, M35.3, M36.0	710.0x, 710.1x, 710.4x, 714.0x, 714.1x, 714.2x, 714.81, 725.x	Rheumatologic disease	1
K25.x-K28.x	531.x, 532.x, 533.x, 534.x	Peptic ulcer disease	1
B18.x, K70.0-K70.3, K70.9, K71.3-K71.5, K71.7, K73.x,	571.2x, 571.4x, 571.5x, 571.6x	Mild liver disease	1

K74.x, K76.0, K76.2-K76.4, K76.8, K76.9, Z94.4			
Main codes: E08, E09, E10, E11, E13. Relevant subcodes: E**.0x, E**.1x, E**.6x, E**.8x, E**.9x	250, 250.0x, 250.2x, 250.3x, 250.7x	Diabetes without chronic complications	1
Main codes: E08, E09, E10, E11, E13. Relevant subcodes: E**.2x, E**.3x, E**.4x, E**.5x	250.4x, 250.5x, 250.6x	Diabetes with chronic complications	2
G04.1, G11.4, G80.0, G80.1, G80.2, G81.x, G82.x, G83.x	334.1x, 342.x	Hemiplegia or paraplegia	2
C0x.x, C1x.x, C2x.x, C30.x-C34.x, C37.x-C41.x, C43.x, C45.x-C58.x, C60.x-C63.x,C76.x, C80.1, C81.x-C85.x, C88.x, C9x.x	140- 172.9, 174-195.8, 200-208.9	Any malignancy including leukemia and lymphoma except malignant nonmelanoma of the skin	2
I85.0x, I86.4, K70.4x, K71.1x, K72.1x, K72.9x, K76.5, K76.6, K76.7	456.0x, 456.1x, 456.2x, 572.2x, 572.3x, 572.4x, 572.8x	Moderate or severe liver disease	3
I12.0, I13.11, I13.2, N18.5, N18.6, N19.x, N25.0, Z49.x, Z99.2	582.x, 583.x, 585.x, 586.x, 588.x	Renal disease, severe	3
B20.x	042.x	HIV infection	3
C77.x-C79.x, C80.0, C80.2	196.x, 197.x, 198.x, 199.0x, 199.1x	Metastatic solid tumor	6
B37.x, C53.x, B38.x, B45.x, A07.2, B25.x, G93.4x, B00, B39.x, A07.3, C46.x, C81-96,	043.x, 044.x	Acquired Immunodeficiency syndrome (AIDS)	6

A31.x, A15-19, B59, Z87.01, A81.2, A02.1, B58.x, R64			

Table S3: ICD-10 CM and ICD-9 CM Codes for primary cancer site diagnosis.

ICD-10 CM codes	ICD-9 CM codes	Cancer site
C00-C14, C30-C33	14.x, 160.x, 161.x	Head and neck
C15	150.x	Esophagus
C16	151.x	Stomach
C18-C20	153.x, 154.x	Colorectal
C17, C21, C24, C26	152.x, 158.x, 159.x	Other GI
C22	155.x	Liver
C23	156.x	Biliary
C25	157.x	Pancreatic
C34	162.x	Lung
C37-C39	163.x, 164.x, 165.x	Mediastinum
C40, C41, C45-C49	170.x, 171.x	Connective Tissue
C42	172.x	Melanoma
C50	174.x, 175.x	Breast
C51-C58	179.x, 180.x, 181.x, 182.x<br>183.x, 184.x	Gynecological
C61	185.x	Prostate
C60, C62, C63	186.x, 187.x	Male genital
C64-C68	188.x, 189.x	Urinary tract
C69-C72	190.x, 191.x, 192.x	Central nervous system
C73	193.x	Thyroid
C74-C75	194.x, 195.x	Other Endocrine
C81	201.x	Hodgkin Lymphoma

C82-C86, C88, C89	202.x	Non-Hodgkin lymphoma
C90	203.x	Multiple Myeloma
C91-C96	204-208.x	Leukemia
C7A	209.x	Neuro-endocrine
